# Quality Evaluation of Walnuts from Different Regions in China

**DOI:** 10.3390/foods12224123

**Published:** 2023-11-14

**Authors:** Xuan Ma, Weijun Wang, Chang Zheng, Changsheng Liu, Ying Huang, Wenge Zhao, Jian Du

**Affiliations:** 1 Hubei Key Laboratory of Lipid Chemistry and Nutrition, Key Laboratory of Oil Seed Processing of Ministry of Agriculture, Oil Crops and Lipids Process Technology National and Local Joint Engineering Laboratory, Oil Crops Research Institute of the Chinese Academy of Agricultural Sciences, Wuhan 430062, China; 82101215472@caas.cn (X.M.);; 2 Aksu Zhejiang Fruit Industry Co., Ltd., Aksu 843000, China

**Keywords:** quality evaluation, different varieties, lipid concomitants, cultivated regions, walnut oil

## Abstract

This study analyzed and evaluated the basic crude fat contents, crude protein contents, phenolic compounds, lipid compositions (fatty acids, phytosterols, and tocopherols), and amino acid compositions of 26 walnut samples from 11 walnut-growing provinces in China. The results indicate that the oil contents of the samples varied from 60.08% to 71.06%, and their protein contents ranged from 7.26 g/100 g to 19.50 g/100 g. The composition of fatty acids corresponded to palmitic acid at 4.61–8.27%, stearic acid at 1.90–3.55%, oleic acid at 15.50–32.28%, linoleic acid at 53.44–67.64%, and α-linolenic acid at 2.45–12.77%. The samples provided micronutrients in widely varying amounts, including tocopherol, phytosterol, and total phenolic content, which were found in the walnut oil samples in amounts ranging from 356.49 to 930.43 mg/kg, from 1248.61 to 2155.24 mg/kg, and from 15.85 to 68.51 mg/kg, respectively. A comprehensive evaluation of walnut oil quality in the samples from the 11 provinces using a principal component analysis was conducted. The findings revealed that the samples from Henan, Gansu, and Zhejiang had the highest composite scores among all provinces. Overall, Yunnan-produced walnuts had high levels of crude fat, polyunsaturated fatty acids, and total tocopherols, making them more suitable for producing high-quality oil, whereas Henan-produced walnuts, although lower in crude fat, had a higher crude protein content and composite score, thus showing the best walnut characteristics.

## 1. Introduction

Walnut (*Juglans regia* L.) is native to the Mediterranean Basin. As an important woody oil plant, it is known as one of the world’s four major nuts, alongside almonds, chestnuts, and cashews [1]. Walnut has a wide distribution range, mainly in eastern Asia, northern Africa, western South America, and southern Europe [2]. As a major walnut-producing country, China ranks first in the world in terms of walnut-planting areas, with its major planting areas being in Xinjiang, Yunnan, Shaanxi, and Hubei. In 2021, China’s total walnut cultivation area reached 745.49 hm^2^, and the total output of walnut (dry weight) was 540.35 million tons [3].

Walnut oil is a potential lipid resource, with walnut kernels containing up to 52–70% oil, containing palmitic, stearic, oleic, linoleic, and linolenic acids, with polyunsaturated fatty acids (PUFAs) being the predominant type of fatty acid [4,5]. The composition and proportion of fatty acids in walnuts largely determine their nutritional value and quality; unsaturated fatty acids (UFAs) are excellent in immune regulation, lipid regulation, and thrombus removal and reduce the risk of cardiovascular and neurological diseases [6,7]. Walnut oil is rich in lipid concomitants, which mainly comprise active ingredients, such as phytosterols, polyphenols, and tocopherols [8]. Phytosterols can be used to treat prostate, arthritis, allergies, and stress-related diseases, as well as to inhibit the occurrence of colon cancer, reduce plasma cholesterols and low-density lipoproteins, and prevent cardiovascular diseases, among other effects [9]. Polyphenols not only have a variety of anti-cancer, anti-aging, antioxidation, and other bioactive functions but also exert a better protective effect on atherosclerosis, the heart, vasodilation, etc. [10]. Zhao et al. [11] showed that walnut kernel extract could increase the activity of antioxidant enzymes and reduce lipid peroxidation products in the serum and tissues of mice. Tocopherol exhibits numerous beneficial properties, such as contributing to reducing the risk of coronary heart disease [12] and having anti-inflammatory and anti-proliferative effects in cancer [13]. A walnut-enriched diet can improve endothelium-dependent vasodilatation in type 2 diabetic individuals [14] and endothelial function in overweight adults with visceral adiposity [15].

The current research on walnuts focuses on the effects of different processing methods and storage processes on the nutritional value and stability of walnut oil, and which different extraction methods have a greater impact on obtaining walnut oil with an nutrients. Gharibzahedi and Koski et al. [13,16] showed that different extraction methods may lead to alterations in the minor components of walnut oil, with antioxidant functions and pro-oxidant effects. In addition to the important influence of different extraction processes, determination methods, and storage methods on the quality of walnut oil, the origin and variety of walnut planting are also important influencing factors. Zhong et al. [17] found that the walnut variety affects the fatty acid composition and content and the bioactive substance content of walnut oil. In China, the nutritional value and quality of walnut oil vary greatly, which is mainly related to the origin of the walnuts and their varieties [18]. The varieties and growing locations of walnuts can lead to variations in the quality of walnut resources, which, in turn, has a significant impact on the processing quality of walnuts [19,20].

Numerous studies have indicated that there are notable variations in the appearance, value, antioxidant activity, and nutritional composition of walnuts originating from different production areas and varieties [19,21]. Moreover, the nutritional quality of walnut oil varies widely due to environmental conditions, like climate and altitude, and China has a wide distribution of walnut cultivation areas [22]. There are various walnut varieties in China, and the main planting varieties in major production areas are relatively fixed due to the geographical environment and government economic support. However, there is a lack of comparative research on walnut oil from different production areas and varieties; thus, the composition characteristics of walnut oils from major production areas are unclear, resulting in difficulties in tracing the varieties of walnut oil from different production areas. China is a major center of walnut genetic diversity, serving as a germplasm source for walnut breeding efforts [23]. The production of walnut oil in China is mainly small-scale and lacks standardized production standards. This has led to various methods of oil extraction, further affecting the compositional characteristics of walnut oil. However, the relative scarcity of large-scale molecular phylogeographic studies of walnuts has made it difficult to accurately determine their native geographic range. Due to the wide range of walnut oil types and varieties, extensive cultivation areas, and diverse processing methods in our country, there is a lack of clarity regarding the compositional characteristics of walnut oil. Therefore, to understand the sources of these differences, it is necessary to analyze the basic physical and chemical properties and nutritional composition of walnuts from different sources. At the same time, a better understanding of the origin, genetic diversity, and structure of Chinese walnuts is needed. It is important to have a comprehensive understanding of the composition and characteristics of walnut oil to guide its production in our country.

High-quality walnut oil cannot be obtained without the use of high-quality raw materials and the selection of specialized varieties. Conducting suitability evaluations by screening for specialized varieties that are suitable for walnut oil processing is also a research direction that deserves significant attention in the future. Thus, this analysis aimed to compare the main components of walnut kernels from different walnut-producing provinces in China (Xinjiang Uyghur Autonomous Region, Gansu Province, Shaanxi Province, Shanxi Province, Hebei Province, Shandong Province, Zhejiang Province, Henan Province, Hubei Province, Sichuan Province, and Yunnan Province). The data were used to estimate and provide a theoretical basis of the nutritional quality, selection, and breeding of good seeds, which could later be used for the deep processing of walnuts from different origins in China; to promote the development of the walnut industry; to determine the fundamental physicochemical characteristics, nutritional composition, and antioxidant capacity of 26 walnut samples sourced from various regions in China; and, lastly, to gain a deeper understanding of the factors that influence the overall quality of walnuts and a more detailed understanding of the processing properties of walnuts from different regions. Comprehending the interplay between these variables and their impact on walnut quality will provide scientific guidance for the processing of high-quality walnut oil and the breeding of walnut varieties.

## 2. Materials and Methods

### 2.1. Sample Collection

**Figure 1:**
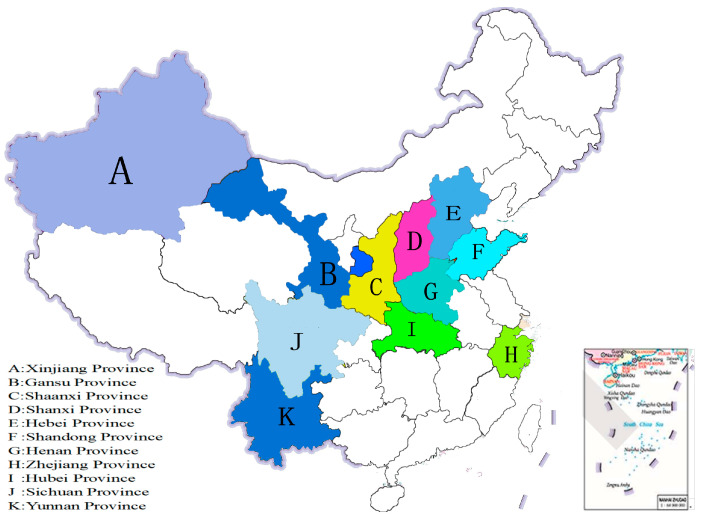
Regional distribution of walnut (*Juglans regia* L.) production in China.

The sampled sites in this study were located in (1) Xinjiang, which is a mountainous terrain and a basin with a temperate continental arid climate; (2) Gansu, which is a mountainous plateau terrain with a temperate monsoon climate; (3) Shaanxi, which is a loess plateau terrain and has a north warm temperate zone climate; (4) Shanxi, which is a mountainous plateau terrain with a temperate continental monsoon climate; (5) Hebei, which is mainly a plain terrain with a temperate monsoon climate; (6) Shandong, which is mainly a plain terrain with a temperate monsoon climate; (7) Zhejiang, which is a hilly terrain with a subtropical monsoon climate; (8) Henan, which has a plain topography and a continental monsoon climate; (9) Hubei, which has a plain topography and a subtropical monsoon climate; (10) Sichuan, which is a basin terrain and has a subtropical humid monsoon climate; and (11) Yunnan, which is a mountainous plateau terrain and has a subtropical monsoon climate. The temperature, annual precipitation, and daylight of these provinces are different. According to data from the National Bureau of Statistics, we selected the top 11 provinces in China in terms of walnut production and sales volume, and we compared their relevant indicators. A total of 26 walnut varieties were obtained during their harvest time (between July and September 2022) from Xinjiang A (WS1, WS2, WS3, WS4), Gansu B (WS5, WS6), Shaanxi C (WS7, WS8, WS9), Shanxi D (WS10, WS11, WS12), Hebei E (WS13, WS14), Shandong F (WS15, WS16), Zhejiang H (WS17), Henan G (WS18), Hubei I (WS19, WS20, WS21), Sichuan J (WS22), and Yunnan K (WS23, WS24, WS25, WS26). The selected plantations are the most important walnut-growing areas (Figure 1), accounting for more than 85% of the walnut-growing areas in China.

### 2.2. Chemicals and Materials

The standards of 40 fatty acid methyl esters, α-tocopherol, β-tocopherol, δ-tocopherol, γ-tocopherol (purity > 95%), 5α-cholestane, stigmasterol, β-sitosterol, fucosterol, cycloarterol, erythrodiol, and gallic acid were purchased from Sigma-Aldrich Chemical Co., Ltd. (Shanghai, China). Methanol, n-hexane, and isopropanol of HPLC grade were purchased from Merck (Darmstadt, Germany). Other reagents and solvents were purchased from Sinopharm Chemical Regent (Shanghai, China).

### 2.3. Analyses of Walnut Kernels

#### 2.3.1. Determination of Protein and Oil Content of Walnut Kernels

The walnut kernels’ oil contents (dry weights), according to GB5009.6-2016 [24], were determined based on a gravimetric analysis using analytical-grade petroleum ether in a *Soxhlet* apparatus (B-811; Buchi-Labortechnik AG) for 18 h. After loading 2–5 g of the samples into pre-weighed filter paper packets, the samples were extracted for 18 h. After extraction, the filter paper packets were dried in an oven at 100 ± 5 °C for 1 h, then weighed, and left in a desiccator for 0.5 h. The above steps were carried out until the difference in mass before and after was ≤2 mg. The oil contents of the walnut kernels were calculated based on the difference between the before and after masses. The protein contents of the walnut kernels were determined using the Kjeldahl method according to GB5009.5-2016 [24]. Briefly, 0.5 g of each sample (dry weight of the crude extract) was weighed and placed into a digestion tube, and Kjeldahl catalytic tablets and 10 mL of concentrated sulfuric acid were added for digestion. After digestion was completed, the digestion tube was placed on a K9860 automatic Kjeldahl nitrogen tester (Haineng Future Technology Group Co., Ltd., Shandong, China) for protein determination, and a conversion factor of 5.3 was used to determine the nut category.

#### 2.3.2. Analysis of Phenolic Compounds in Walnut Kernels

The extraction of phenolic compounds from the walnut kernels was performed according to Cong et al. [25]. Briefly, 0.5 g of crushed walnut samples was weighed, and a methanol–water mixture with 70% methanol (7:3, *v*/*v*) was added. During the extraction process, the samples were shaken by a vortex mixer at 2500 rpm for 20 min and centrifuged at 4863× *g* for 10 min, and the extraction process was performed three times with the extracted liquids. The phenolic compounds in the walnut samples were identified and quantified using liquid chromatography–mass spectrometry based on target metabolomics, and the instrument parameters were set according to Zhang et al. [26]. The chromatographic separation of phenolic compounds was achieved with a C18 Zorbax Eclipse Plus column (4.6 × 100 mm i.d., 1.8 μm) (Agilent, Santa Clara, CA, USA) at a column oven temperature of 40 °C. Mobile phases A and B were water and acetonitrile containing 0.1% (*v*/*v*) acetic acid, respectively, and the injection volume was 5 µL. A 20 min gradient process was used for the separation of phenolic compounds at a flow rate of 0.4 mL·min^−1^. The percentages (*v*/*v*) of solvent B were as follows: 0–2 min at 5%, 2–4 min at 5% to 15%, 4–10 min at 15% to 60%, 10–15 min at 60% to 95%, 15–16 min at 95%, 16–18 min at 95% to 60%, and 18–20 min at 60% to 5%, followed by 2 min of re-equilibration at 5% before the next injection. The ion source parameters were set as follows: ion source temperature at 550 °C, ion spray voltage at 4.5 kV, curtain gas at 35 mL·min^−1^, nebulizer gas at 40 mL·min^−1^, and heater gas at 45 mL·min^−1^. The optimization of the molecular deagglomeration potential (DP) and collision energy (CE) was carried out via direct injection using liquid chromatography–mass spectrometry (LC-MS). Analyst 1.6 software (AB Sciex, Framingham, MA, USA) was used for data acquisition and processing.

### 2.4. Analyses of Walnut Oils

#### 2.4.1. Lipid Extraction

The walnut shells were manually peeled off, and the manually peeled walnut shells were crushed using a crusher (FW80, Tianjin Teste Co., Ltd., Tianjin, China), while the walnut kernels were cut into pieces of about 3 mm in size. Then, 5% of the crushed walnut shells was added to the walnut kernels, and the two were mixed well together. The kernels were pressed using a cold press (CA59, Monchengladbach, Germany) and centrifuged at 4863× *g* for 30 min (Sorvall Stratos, Thermo Fisher Co., Ltd., Waltham, MA, USA). All samples were placed in amber glass bottles, which were then sealed and stored in a refrigerator at 4 °C until analysis.

#### 2.4.2. Determination of Physical and Chemical Properties

The acid value (AV) and peroxide value (PV) were determined based on the AOCS Official Method cd 3d-63 [27] and the AOCS Official Method cd 8–53 [28], respectively, using the titration method. The iodine value (IV) was determined based on the GC data shown in Table S1, according to Chira et al. [29] and BS EN 16300:2012 [30].

The calculation formula for IV was as follows:

(1) $$Ci=\frac{25,381\times Ni}{Mi}\;IV\;(g\;I2/100\;g\;oil)=\sum_{i}Ui\times Ci$$

where 253.81 is the molecular weight of the iodine molecule (I2); Ni is the number of olefinic double bonds in the fat component; Mi is the molecular mass of the fat component; and Ui is the content of individual fat components, given in %.

#### 2.4.3. Fatty Acid Composition

The determination of the fatty acid content was performed according to GB5009.168-2016 [24]. An oil sample of 65 mg was dissolved in 2.5 mL of n-hexane and mixed with 0.5 mL of 0.5 mol/L NaOH-CH_3_OH at a speed of 2500 rpm/min for 3 min. After the mixture was centrifuged at 4863× *g* for 5 min, the supernatant was extracted to prepare fatty acid methyl esters (FAMEs). The fatty acid content was analyzed using a 7890 A gas chromatograph (GC) (Agilent, Santa Clara, CA, USA) equipped with a flame ionization detector (FID) for detecting fatty acid methyl esters. The column was DB-FFAP (30 m × 250 µm id × 0.25 µm thickness). The detailed operation conditions were as follows: nitrogen was the carrier gas; the injector and detector temperatures were 250 °C and 280 °C, respectively, and the flow rate of nitrogen was 1.5 mL/min with a split ratio of 80:1; the oven temperature was 130 °C (kept for 3 min), and this was increased to 200 °C (at a rate of 5 °C/min and held for 10 min) and finally to 220 °C (at a rate of 2 °C/min and held for 3 min); and the total run time was 40 min. Fatty acids were identified by comparing the retention times with the standards of 40 fatty acid methyl esters, and their levels are reported in terms of the relative proportions.

#### 2.4.4. Tocopherols

The total tocopherol content (TTC) of the oil samples was measured according to the AOCS Official Method Ce8-89 [31], with some modifications. Approximately 2 g of extracted oil was dissolved in 25 mL of n-hexane and mixed. Then, 1.2 mL of the mixture solution was filtered through a 0.22 µm nylon syringe filter and directly injected into an injection vial for analysis. The HPLC conditions were the same as those reported by Cong et al. [25]. A diode array detector (SPDM20A, Shimadzu, Tokyo, Japan) was used to detect resorcinol at specific wavelengths. The concentration was determined using ultraviolet spectroscopy based on Beer’s law (λ = 292 nm, 298 nm, and 298 nm). The flow rate was 1.0 mL/min, and the mobile phase was a mixture of hexane and isopropanol (0.5:99.5, *v*/*v*). Each tocopherol in the analyzed oil samples was identified according to the retention times of reference samples of tocopherols in the chromatogram.

#### 2.4.5. Phytosterol

To saponify 600 mg of the walnut oil, we added 0.5 mL of 0.5 mg/mL 5-α-cholestane as the internal standard, and then 10 mL of 2 mol/L KOH-C_2_H_5_OH was added to the sample. The oil samples were placed in a saponified bath at 60 °C for 60 min. After saponification was completed, we added 4 mL of distilled water and 10 mL of hexane to the samples, rotated them at 2500 rpm for 3 min, and then transferred the supernatant to another centrifuge tube. The extraction was repeated three times. Following this, a small amount of anhydrous sodium sulfate was added to the centrifuge tube to draw out excess water. Then, we transferred the samples to 15 mL glass tubes and baked them in an 85 °C oven until dry. The extracted unsaponifiable matters were silylated with 100 µL of BSTFA + TMCS (N, O-Bis (trimethyisilyl) trifluoroacetamide with trimethylchlorosilane) for detection, and the mixture was allowed to react in an oven at 105 °C for 15 min. Finally, we used n-hexane to increase the volume to 1 mL and added it to a glass tube containing the injection bottle for analysis. The working conditions were the same as those detailed by Wang et al. [32].

#### 2.4.6. Polyphenols

The polyphenol content of the walnut oil was determined using the Folin–Ciocalteu colorimetric method with a few modifications [25]. Briefly, 1.25 g of the walnut oil was added to 1.5 mL of hexane and 1.5 mL of an 80% methanol–water solution (8:2, *v*/*v*). During the extraction process, the samples were shaken by a vortex mixer at 2500 rpm for 5 min and centrifuged at 4863× *g* for 10 min; the extraction process was repeated three times, and the extracted liquids and the extracts were combined to obtain the polar extract of walnut oil. A total of 0.5 mL of the polar extract of the walnut oil was mixed with 5 mL of distilled water and 0.5 mL of the Folin–Ciocalteau phenol reagent, equilibrated for 3 min, mixed with 1.0 mL of a clarified saturated sodium carbonate solution, and then fixed to 10 mL with distilled water. Following a 60 min reaction in the dark, the absorbance was measured at 765 nm using a UV/visible spectrophotometer (DU800, Beckman Coulter, Inc., Brea, CA, USA). SA was used for calibration, and the results are expressed as gallic acid equivalents (mg GAE/kg).

#### 2.4.7. Oxidative Stability Index and Free Radical Scavenging Capacity Assays

The oxidative stability was evaluated, and the stability is expressed as the oxidation induction period (IP); it was measured using a Rancimat 743 (Metrohm, Riverview, FL, USA). A total of 3.0 g of the oil sample was heated to 110 °C under an air flow of 20 L/h. The conductivity of the water was measured automatically as oxidation proceeded, and an oxidation curve was generated by plotting the conductivity as a function of time produced, whose point of inflection is known as the induction time, and the result was recorded in hours [33].

The 2,2-diphenyl-1-picrylhydrazyl (DPPH) and ferric-reducing antioxidant power (FRAP) were measured following the method of Cong et al. [24]. The DPPH and FRAP results are expressed as micromoles of Trolox equivalents per 100 g of sample (µmol TE/100 g).

### 2.5. Analyses of Walnut Cakes

The determination of hydrolyzed amino acids was performed according to GB 5009.124-2016 [24]. The sample was accurately weighed with 0.1 g of walnut cakes and 6 mol/L of concentrated hydrochloric acid in a hydrolysis tube, filled with nitrogen to remove air from the tube, and sealed. Then, the tube was hydrolyzed in a 105 °C hydrolysis oven for 24 h, removed, and cooled to room temperature. The hydrolysate was filtered into a 50 mL volumetric flask, the hydrolysis tube was rinsed several times with distilled water, and the volume was fixed. A total of 1 mL of the filtrate was weighed accurately, placed in a 25 mL beaker, and evaporated in a 100 °C water bath. Then, 5 mL of distilled water was added to fix the volume, and the filtrate was passed through a 0.22 µm filter membrane and then placed on a machine (Hitachi LA8080 Hitachi High-Tech Corporation, Tokyo, Japan) for determination. The instrument parameters were the same as those used by Han et al. [34].

### 2.6. Statistical Analysis

The results are shown as the mean ± standard deviation (SD) of three replicates. A statistical analysis was carried out using SPSS 23.0 (IBM, Armonk, NY, USA) software packages. For all evaluated parameters (Duncan’s multiple-range tests), the ANOVA test results were considered to be significantly different at the 5% level. A correlation analysis, cluster analysis, and principal component analysis were carried out using Origin 9.0 software (Origin Lab Corp., Northampton, UK).

## 3. Results and Discussion

### 3.1. Crude Protein and Crude Fat in Walnut Kernels

**Figure 2:**
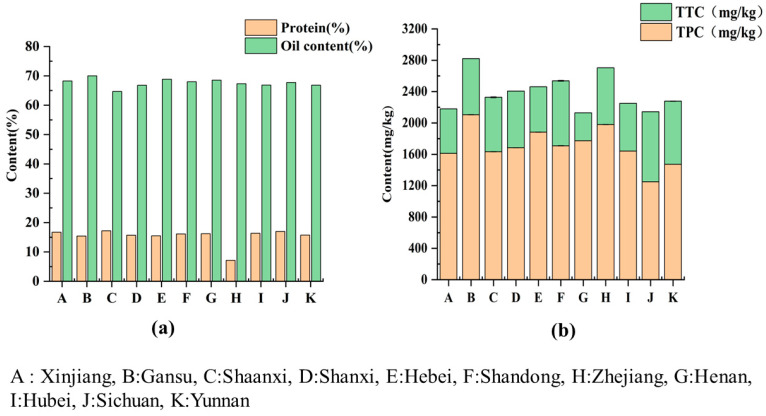
(**a**) The crude fat and crude protein in walnuts from 11 walnut-planting provinces in China. (**b**) Total tocopherol contents and total polyphenol in walnuts from 11 walnut-growing provinces in China.

Protein, as an important material base of the human body, plays an important role in various metabolic activities of the human body. A statistical analysis was performed to determine the protein and oil contents of 26 varieties of walnut kernels. As shown in Figure 2a, the crude protein contents were significantly different across the 11 provinces. The average content of crude protein was up to 17.20% in the walnut kernels from Shaanxi, and the highest crude protein content was obtained in Shaanxi-WS9 (19.5 g/100 g), while the lowest was obtained in Zhejiang WS17 (7.26 g/100 g). The difference in the average crude protein content of the walnuts from Shandong, Henan, and Hubei was small. Zhao et al. [35] reported that the crude protein content of walnuts from Yunnan Province was about 15% and that the crude protein content of walnuts from Shanxi was more than 15%; this is consistent with our findings. However, the crude protein content of the Xinjiang walnuts obtained by the study (16.70%) is lower than that previously reported (19.35%). The protein content of these Chinese walnut kernels is similar to that reported by Sze-Tao and Sathe (16.66%) [36] but slightly lower than that reported by Kafkas (13.57–25.72%) [37]. The content and quality of the protein in walnuts are closely related to the species and environmental factors, including season, temperature, moisture, and light.

As an important part of the human body, fat plays a crucial role in human body function. The oil content is an important indicator for evaluating walnut kernels, and the oil contents of walnuts of different origins and varieties vary. The amount of crude fat was measured in the 26 walnut kernel samples. As shown in Figure 2b, it was found that there were significant differences in the crude fat content of the walnut kernels across the 11 provinces. The crude fat content of the walnuts from Gansu (70.00%) was a little higher than that in the walnuts from Henan (68.52%) and Xinjiang (68.27%). Among the different types of walnuts, Shaanxi-WS7 had the lowest content of crude fat (62.34%), and Yunnan-WS23 had the highest content of crude fat (71.06%). Compared to other typical oil crops, the oil contents of walnut kernels are much higher than those of hemp (26.25–37.50%) [38], rapeseed (35.00–39.00%) [39], and peanuts (45.97–57.28%) [40]. Poggetti et al. [41] found that the oil contents of walnuts showed a significant positive correlation with temperature. Crews et al. [42] compared the oil content of walnuts from seven different countries and found that the differences in oil content were mainly due to geography. Different walnut cultivation areas vary greatly in the oil contents produced; therefore, the main factor affecting crude fat may be the origin of walnut cultivation.

### 3.2. Phenolic Compounds in Walnut Kernels

**Figure 3:**
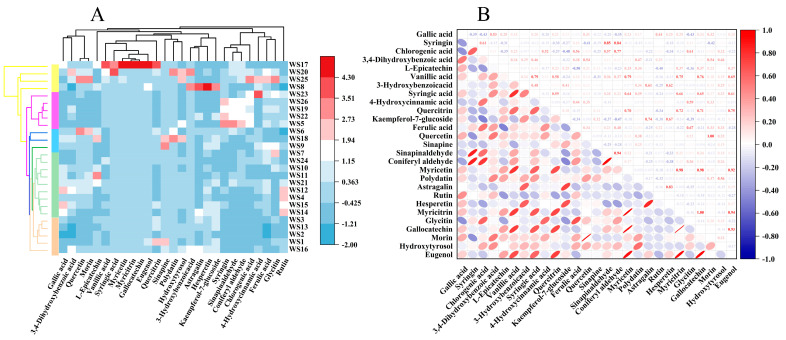
(**A**): Hierarchical clustering heatmap analysis of phenolic compounds in walnut kernels. (**B**): Correlation heatmap of phenolic compounds in walnut kernels.

The phenolic compounds in walnuts are mainly hydrolyzable tannins and phenolic acids, whose percentages can vary depending on the walnut variety, with the former accounting for 60–80% and flavonols accounting for 26–35% [43]. In this study, the content and composition of the phenolic compounds in all walnut samples were analyzed. Three major classes of compounds were detected in all walnut samples, namely, phenolic acids, tannins, and flavonoids, which included a total of 27 types of phenolic compounds, such as gallic acid, syringin, chlorogenic acid, and 3,4-dihydroxybenzoic acid. This study found that the phenolic compounds present in the walnut kernels from different origins were mainly gallic acid, syringin, chlorogenic acid, quercitrin, ferulic acid, rutin, glycitin, gallocatechin, morin, myricetin, and quercetin. Among them, gallic acid had the highest content. Wu et al. [44] reported that walnut kernels contained significant amounts of ellagic acid, gallic acid, catechin, ferulic acid, epicatechin, and syringic acid, and ellagic acid was the most important antioxidant in the kernel, accounting for more than 20%, 40%, and 15% of antioxidants. The differences in phenolic compounds and their contents among different samples may be explained by the variations in the total phenolic content and key antioxidants, as well as origin and variety. The distribution of the phenolic compounds in all walnut kernel samples was evaluated using a cluster heatmap analysis, and the results are shown in Figure 3A. The relative abundance of each phenolic compound was normalized and visualized using color depth. The heatmap intuitively reflects the relative abundance of the phenolic compounds accumulated in the different walnut kernel samples. Based on the accumulation pattern of the phenolic compounds in the walnut kernels, the samples can be divided into five different clusters.

Among them, the phenolic compounds in the first cluster included vanillic acid, 3-hydroxybenzoicacid, syringic acid, 4-hydroxycinnamic acid, sinapinaldehyde, coniferyl aldehyde, and polydatin, but eugenol accumulated in smaller amounts in the WS1, WS2, WS3, WS13, and WS16 walnut varieties. The phenolic compounds of the second cluster were mainly distributed in the WS4, WS10, WS11, WS12, WS14, WS15, WS21, and WS24 varieties and mainly included gallic acid, syringin, chlorogenic acid, 3,4-dihydroxybenzoic acid, quercitrin, kaempferol-7-glucoside, ferulic acid, quercetin, coniferyl aldehyde, myricetin, glycitinand, gallocatechin, morinand, and hydroxytyrosol. The phenolic compounds of the third cluster were mainly distributed in WS6, WS9, and WS18, with less accumulation or no accumulation of 3-hydroxybenzoicacid, syringic acid, sinapinaldehyde, astragalin, myricitrinand, and eugenol. The phenolic compounds of the fourth cluster mainly accumulated in WS5, WS19, WS22, WS23, and WS26, with outstanding performance of syringin, sinapinaldehyde, and coniferyl aldehyde, which showed less accumulation in the other clusters. The fifth cluster’s phenolic compounds primarily accumulated in the WS8, WS17, WS20, and WS25 varieties, with outstanding percentages of L-epicatechin, vanillic acid, myricetin, quercitrin, myricitrinand, and gallocatechin.

The correlations between the different phenolic compounds were further studied using a heatmap (Figure 3B). Among the major compounds, gallic acid and syringin showed a negative correlation, while chlorogenic acid, coniferyl aldehyde, and glycitin also showed negative correlations. It is noteworthy that syringin showed positive correlations with chlorogenic acid, sinapinaldehyde, and coniferyl aldehyde but negative correlations with other phenolic compounds. Quercetin and morin were strongly correlated with each other but weakly or not at all correlated with other substances. In summary, there are significant differences in the phenolic compounds in the walnut kernels, and the correlations between the different phenolic compounds vary among the different walnut kernels. Additionally, there are significant differences in the presence of different phenolic compounds in the walnut kernels. These results provide some guidance on the phenolic composition and distribution of different walnut varieties from five regions in China.

### 3.3. Physicochemical Analysis of Walnut Oils

An analysis of physical and chemical properties is one of the important requirements for evaluating the quality of oils and fats. By testing and analyzing these indicators, it is possible to determine whether oils and fats are affected by oxidation or other adverse reactions and to obtain information about their stability and suitability. The quality indicators were measured for all walnut samples from different regions in China, and the results are shown in Table 1. There were significant differences in the AV of all samples of walnut oil. The Shaanxi-WS10 and WS12 walnut oil samples had the lowest (0.139 mg/g) and the highest AV (0.77 mg/g), respectively. The AV of the samples met the first-class standard (≤1.0 mg/g) in the GB/T 22327-2019 “Walnut Oil” specification [45] (SPC, 2019). The peroxide value (PV) is an important indicator for determining the oxidation of fats and oils. The PV in the walnut oils of Henan-WS18 was 0.00 g/100 g, whereas that in the walnut oils of Xinjiang-WS1 was 0.02 g/100 g. Previous research has shown that there is a relationship between PV and the composition of fatty acids, as well as the content of phenolic compounds and other active substances, in oils and fats [46]. The PV of all samples complied with the national standard for the food safety of plant oils, GB 2716-2018 (≤0.25 g/100 g) [47] (SPC, 2018). IV is a relevant quality descriptor, as it reflects the global unsaturation of a sample and allows for a reliable comparison of samples in terms of unsaturation. The higher the degree of unsaturation, the more the double bonds present in fatty acids, making fats less stable and more prone to oxidation. There was a significant difference in IV among samples from different origins, with the highest IV of 145.64 g I2/100 g oil being found in WS25 from Yunnan and the lowest IV of 162.14 g I2/100 g oil being observed in WS21 from Hubei. A lower IV may be indicative of the presence of few unsaturated bonds and would certainly indicate less unsaturated fatty acids. It has been reported that the environment, genotype, nut maturity, and their interactions all contribute significantly to the variations in the degree of unsaturation of walnut oil [48].

#### 3.3.1. Fatty Acid Composition

The fatty acid composition of walnut oil is of great reference value for evaluating its economic and nutritional value. Walnuts of different origins may have the same fatty acid composition but significant variations in their content. Thus, the main fatty acid compositions and contents of the 26 walnut oil samples were measured, and the results are shown in Table S1. The fatty acid composition mainly corresponded to palmitic acid from 4.61 (Yunnan-WS25) to 8.27% (Zhejiang-WS17), stearic acid from 1.90 (Yunnan-WS26) to 3.55% (Hebei-WS14), oleic acid from 15.50 (Hubei-WS21) to 32.28% (Yunnan-WS24), linoleic acid from 53.44 (Yunnan-WS24) to 67.64% (Yunnan-WS26), and α-linolenic acid from 2.45 (Yunnan-WS26) to 12.77% (Shandong-WS16). There were significant differences in the composition and content of each fatty acid across the different samples from the various regions (*p* <0.05). Gharibzahedi [46], Hu [49], Zhang [50], and Yu et al. [51] found significant differences in the fatty acid composition of different walnut varieties from different origins and of the same origin, which is consistent with our findings. Walnut oils are a great source of UFAs (from 89.36 in Gansu-WS6 to 93.21% in Yunnan-WS25). Although UFAs have many benefits, they are prone to lipid peroxidation; this produces substances such as free radicals and reactive oxygen species, which can cause certain damage to cells and tissues. Crews et al.’s [42] comparison of the fatty acid composition of walnut oil from seven different countries revealed differences in the fatty acid composition and content due to geographical variations. This may explain why certain differences were found in this study in the fatty acid composition of the walnuts grown in different environments. Greve et al. [52] proposed the interaction of genetic factors to explain the differences in the content of lipid components across different varieties. It has been shown that the planting environment, type, and variety of walnut have a significant impact on the composition and content of walnut oil fatty acids, regardless of whether it is cultivated in different producing areas in China or in different countries.

#### 3.3.2. Oxidative Stability Index and Free Radical Scavenging Capacity

The differences in the antioxidant capacity of raw materials may be related to the types and contents of polyphenols, the fatty acid composition, the phytosterols, and the other trace components in oils. The process of IP refers to the accelerated oxidation of grease at high temperatures under an excessive amount of air. The oxidative stability results of the walnut oil samples at 110 °C are shown in Table 1. The IP (h) of the walnut oil from different regions ranges from 2.53 h (Shanxi-WS13) to 8.41 h (Zhejiang-WS17), and there are significant differences in IP among the different regions (*p* < 0.05). According to Bujdosó et al. [48], the correlation between an oil’s induction time and the PUFA content is negative, so the induction time increases as the linoleic and linolenic acid contents decrease, and our results show the same tendency. The difference in IP is not only related to the origin and variety but also has some correlation with the fatty acid composition and trace nutrient content of samples.

The results of the free radical scavenging ability of the polar extracts from non-local walnut oil measured using 1,1-diphenyl-2-picrylhydrazyl (DPPH) and ferric-reducing antioxidant power (FRAP) are shown in Table 1. The results indicate that DPPH had a stronger scavenging ability for Gansu-WS6 (42.31 µmol/100 g) and a weaker scavenging ability for Shandong-WS16 (7.44 µmol/100 g), while FRAP had a stronger scavenging ability for Hubei-WS21 (94.15 µmol/100 g) and a weaker scavenging ability for Shandong-WS16 (24.22 µmol/100 g). The capacities of DPPH and FRAP to scavenge free radicals in WS16 were relatively weaker. In general, there were certain differences between the results of the free radical scavenging capacity and oxidative stability, which might be related to the environments in which the varieties originated from and their genetics.

#### 3.3.3. Tocopherol Content

The contents of three homologs (α-, γ-, and δ-tocopherol) of tocopherols in the walnut oil samples were studied, as shown in Table S1. The results show that the TTC in all walnut oil samples ranged from 356.49 (Henan-WS18) to 930.43 mg/kg (Yunnan-WS23). Among them, the content of γ-tocopherol was the highest, ranging from 298.30 (Henan-WS18) to 707.45 mg/kg (Sichuan-WS22), which accounted for more than 70% of the total tocopherol content. It is worth noting that δ-tocopherol was not detected in the Henan-WS18 samples. As shown in Figure 2b, the average total tocopherol content of the Sichuan walnuts was the highest at 894.16 mg/kg, followed by the average total tocopherol contents of the Shandong and Yunnan walnuts at 829.50 mg/kg and 805.38 mg/kg, respectively, while the Henan walnuts had the lowest average total tocopherol content at 356.49 mg/kg. Overall, there were significant differences in the tocopherol content of the different varieties of walnut oil. The total tocopherol content of the walnut oil from China is higher than that reported in walnut oils from Argentina (247.00–365.00 mg/kg) [53], Spain (186.50–436.20 mg/kg) [54], and Canada (128.42–307.98 mg/kg) [55]. Gharibzahedi et al. [46] compared the content of tocopherols in ordinary walnut oil obtained from three different varieties of walnuts and proved that the variety of walnut affects the content of tocopherols in its oil. As described by Lavedrine et al. [56], geographic location is one of the main factors affecting the tocopherol content of walnuts. The main factors responsible for the significant variability in the tocopherol content may be related to the origin of walnut cultivation and its environment and walnut genetic variability, which explains the results regarding the variability in the tocopherol content quite well.

#### 3.3.4. Phytosterol Content

Phytosterols, as the main component of the unsaponifiable substances in many oils, are generally considered nutritional supplements for fats and oils. As shown in Table S1, among all samples, the phytosterol content (TPC) ranged from 1248.6 mg/100 g (Sichuan-WS22) to 2155.24 mg/kg (Gansu-WS5). The contents of the five phytosterols were as follows: stigmasterol ranged from 31.60 (Sichuan-WS22) to 108.80 mg/kg (Shaanxi-WS8), β-sitosterol ranged from 930.80 (Yunnan-WS26) to 1471.60 mg/kg (Hubei-WS20), fucosterol ranged from 67.40 (Yunnan-WS26) to 443.2 mg/kg (Zhejiang-WS17), cycloartenol ranged from 36.70 (Shaanxi-WS8) to 359.90 mg/kg (Shandong-WS16), and erythrodiol ranged from 0 to 607.20 mg/kg (Gansu-WS6). β-sitosterol was the main phytosterol found in the walnut oil samples, accounting for more than 50% of the total phytosterol content. It is worth noting that erythrodiol was only detected in Gansu-WS5, Gansu-WS6, Shaanxi-WS7, Hebei-WS14, and Zhejiang-WS17. The statistical analysis showed significant differences (*p* < 0.05) in the TPC of the walnut oils from different origins. As shown in Figure 2b, the average total phytosterol content of the Gansu walnut oil samples was the highest at 2104.50 mg/kg, followed by the average total phytosterol contents of the Zhejiang and Hebei walnut oil samples at 1979.10 mg/kg and 1882.13 mg/kg, respectively, while the Sichuan walnut oil samples had the lowest average total phytosterol content at 1248.60 mg/kg. The TPC averages (1594.07 mg/kg) obtained from these walnut oil samples are higher than those reported by Gao et al. [57]. The TPC of Chinese walnut oil is similar to that reported in Portugal, which ranges between 1271.00 and 2026.00 mg/kg [58]. Gao et al. [18] showed that there was significant variability in the TPC of different varieties of walnut oil from different origins, and the TPC of iron walnut oil was lower than that of common walnut oil. They revealed that there were significant differences in the TPC of walnut oil from different origins and varieties. It is worth noting that there were certain differences in the TPC of the walnut oil samples from different origins, but these differences were not significant. Therefore, the unique composition of plant sterols can serve as a fingerprint for identifying walnut oil.

#### 3.3.5. Polyphenol Content

Anderson’s research showed that the polyphenols in walnut oil have good inhibitory effects on the oxidation of plasma and low-density lipoprotein outside the body [59]. The determination results of the polyphenol content of the walnut oil samples are shown in Table S1. There were significant differences in the polyphenol content of the walnut oils from different origins (*p* < 0.05). Among all samples, the polyphenol content of the walnut oil samples ranged from 15.85 mg/kg (Shandong-WS16) to 68.51 mg/kg (Xinjiang-WS4), showing significant differences in the polyphenol content of the different samples. The polyphenol content of the walnut oil samples in this study is lower than that in the study by Gao et al. (33.45–84.49 mg/kg) [57]. Gao et al. [18] found that there were significant differences in the polyphenol content of different varieties of walnut oil, and the polyphenol content of iron walnut ranged from 9.42 to 23.94 mg/kg, while that of common walnut ranged from 44.78 to 64.61 mg/kg. Overall, the level of polyphenol content of Chinese walnut oil is relatively low compared to that of other oils [25,26]. This suggests that the origin and variety are the main factors affecting the polyphenol content of walnut oil.

### 3.4. Comprehensive Score Analysis

The principal component analysis (PCA) is a dimensionality reduction statistical method that compresses multiple original datapoints into several principal components, which can represent the information of the original variables while maintaining the features with a maximum contribution to variance among the samples. PCA was used to evaluate the quality of walnut oil from 11 provinces in China based on nutritional components (phytosterol composition, tocopherol composition, fatty acids, and total phenolic content) and oxidative stability (AV, PV, DPPH, FRAP, and IP). The results showed that the cumulative variance contribution rate for the first five principal components was 83.71% (PC1 = 29.40%, PC2 = 54.92%, PC3 = 66.68, PC4 = 76.37, and PC5 = 83.71%; Table 2). PC1 mainly included palmitic acid (C16:0), SFA, fucosterol, DPPH, IP, and FRAP (principal component contents of 0.92, 0.82, 0.83, 0.47, 0.91, and 0.74, respectively); PC2 mainly included stearic acid (C18:0), linoleic acid (C18:2), α-linolenic acid (C18:3), stigmasterol, β-sitosterol, and cycloarterol (principal component contents of 0.67, 0.75, 0.82, 0.43, 0.51, and 0.79, respectively); PC3 mainly included δ-tocopherol, PV, and the total phenol content (principal component contents of 0.41, 0.52, and 0.85, respectively); PC4 mainly included the oil content (0.71); and PC5 mainly included erythrodiol (0.81). Therefore, the five principal components could replace the original 23 indicators to evaluate the nutritional quality of the walnut oil samples of different origins.

Based on the eigenvalues and principal component loadings shown in Table 3, the functional expressions for the five principal components were obtained as follows:

P1 = −0.34X_1_ + 0.05X_2_ + 0.35X_3_ − 0.18X_4_ − 0.10X_5_ + 0.08X_6_ − 0.06X_7_ + 0.31X_8_ − 0.31X_9_ + 0.14X_10_ + 0.11X_11_ − 0.03X_12_ − 0.25X_13_ + 0.01X_14_ + 0.32X_15_ − 0.20X_16_ + 0.12X_17_ + 0.11X_18_ − 0.08X_19_ + 0.18X_20_ + 0.35X_21_ + 0.04X_22_ + 0.28X_23_.

P2 = −0.039X_1_ + 0.177X_2_ − 0.033X_3_ + 0.28X_4_ − 0.368X5 + 0.31X_6_ + 0.34X_7_ + 0.10X_8_ − 0.11X_9_ + 0.04X_10_ − 0.30X_11_ − 0.28X_12_ + 0.18X_13_ + 0.21X_14_ + 0.05X_15_ + 0.33X_16_ + 0.10X_17_ − 0.29X_18_ − 0.21X_19_ + 0.18X_20_ − 0.04X_21_ + 0.02X_22_ + 0.10X_23_.

P3 = −0.006X_1_ + 0.09X_2_ + 0.13X_3_ + 0.17X_4_ − 0.033X_5_ − 0.25X_6_ + 0.26X_7_ + 0.22X_8_ − 0.22X_9_ + 0.24X_10_ − 0.10X_11_ + 0.25X_12_ + 0.04X_13_ − 0.05X_14_ + 0.08X_15_ + 0.03X_16_ − 0.03X_17_ − 0.12X_18_ + 0.32X_19_ − 0.33X_20_ + 0.02X_21_ + 0.52X_22_ − 0.30X_23_.

P4 = 0.18X_1_ + 0.48X_2_ + 0.13X_3_ − 0.10X_4_ − 0.07X_5_ + 0.09X_6_ − 0.03X_7_ + 0.10X_8_ − 0.10X_9_ − 0.40X_10_ + 0.11X_11_ − 0.21X_12_ − 0.32X_13_ + 0.12X_14_ − 0.29X_15_ + 0.05X_16_ + 0.22X_17_ + 0.16X_18_ + 0.35X_19_ − 0.04X_20_ − 0.20X_21_ + 0.13X_22_ + 0.06X_23_.

P5 = 0.16X_1_ + 0.16X_2_ + 0.03X_3_ − 0.15X_4_ + 0.08X_5_ − 0.18X_6_ + 0.09X_7_ − 0.04X_8_ + 0.04X_9_ + 0.28X_10_ + 0.005X_11_ + 0.20X_12_ + 0.23X_13_ − 0.31X_14_ − 0.14X_15_ − 0.10X_16_ + 0.62X_17_ − 0.20X_18_ − 0.04X_19_ + 0.36X_20_ − 0.15X_21_ + 0.03X_22_ + 0.14X_23_.

X_1_ = protein; X_2_ = oil content; X_3_ = C16:0; X_4_ = C18:0; X_5_ = C18:1; X_6_ = C18:2; X_7_ = C18:3; X_8_ = SFA; X_9_ = UFA; X_10_ = α-tocopherol; X_11_ = γ-tocopherol; X_12_ = δ-tocopherol; X_13_ = stigmasterol; X_14_ = β-sitosterol; X_15_ = fucosterol; X_16_ = cycloarterol; X_17_ = erythrodiol; X_18_ = AV; X_19_ = PV; X_20_ = DPPH; X_21_ = IP; X_22_ = total phenols; X_23_ = FRAP.

*p* = 0.31P1 + 0.27P2 + 0.12P3 + 0.10P4 + 0.08P5.

Finally, comprehensive scores of the 11 walnut-producing regions were calculated based on the composite evaluation function. The higher the comprehensive score, the better the nutritional quality of the walnut oil from that region. As shown in Table 3, Henan-produced walnut oil had the highest comprehensive score of 2.06 points, while Yunnan-produced walnut oil had the lowest score of −1.78 points. A comparison of all samples revealed that WS24 from Yangbi County in Yunnan had the highest PUFA content, while WS23 from Dayao County in Yunnan had the highest total tocopherol content.

### 3.5. Amino Acid Composition in Walnut Cakes

**Figure 4:**
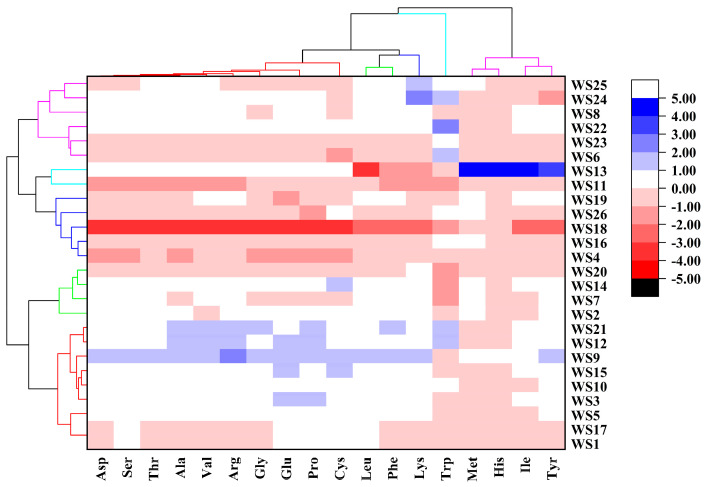
Hierarchical clustering heatmap analysis of amino acid content of walnut cakes.

The amino acid composition is an important chemical property of walnut proteins because it reflects the protein content or the proportion of protein of walnut. A statistical analysis was performed to analyze the hydrolyzed amino acid composition and content of 26 walnut cake samples. The amino acid composition of the walnut cake samples was elucidated using a heatmap analysis. The results were analyzed using the “Euclidean” clustering method to better understand potential regional differences. The relative abundance of each amino acid was normalized and visualized using color depth. As shown in Figure 4, the amino acid contents of the walnut cakes from different origins varied significantly. According to the accumulation patterns of the amino acids in the walnut cakes, the samples could be divided into different clusters corresponding to different walnut varieties. Among them, the first cluster mainly included aspartic acid, serine, threonine, alanine, valine, arginine, glycine, glutamic, proline, and cysteine, which accumulated in the WS1, WS3, WS5, WS9, WS10, WS12, WS15, WS17, and WS21 walnut cake samples. The second cluster mainly included leucine and phenylalanine, which accumulated in the WS2, WS7, WS14, and WS20 walnut cake samples, and lysine mainly accumulated in the WS4, WS16, WS18, WS19, and WS26 walnut cake samples. Tryptophan accumulated prominently in cluster 4 (WS11 and WS13), while the amino acids in cluster 5, including methionine, histidine, isoleucine, and tryptophan, mainly accumulated in the WS6, WS8, WS22, WS23, WS24, and WS25 samples.

It is worth noting that, except for in WS11 and WS24, methionine was detected in all of the other 24 samples; meanwhile, histidine was detected in all samples. Therefore, except for WS11 and WS24, the other 24 samples contained the eight essential amino acids required by the human body (threonine, valine, lysine, isoleucine, leucine, phenylalanine, tryptophan, and histidine). In addition to these essential amino acids, all 26 walnut samples contained eight non-essential amino acids (aspartic acid, serine, glutamic acid, proline, glycine, alanine, histidine, and arginine) and two semi-essential amino acids (cysteine and tyrosine).

## 4. Conclusions

The results of this study indicate that there are differences in the compositions and contents of the crude fat, crude protein, phenolic compounds, lipid components (fatty acids, plant sterols, and tocopherols), and amino acids, as well as the oxidative stability (AV, PV, DPPH, FRAP, and IP), of the walnut samples obtained from different growing environments. The results show that the oil content of the samples ranged from 60.08% to 71.06%, and the protein content ranged from 7.26 g/100 g to 19.50 g/100 g. In addition, the walnut oil samples also had varying amounts of trace nutrients, including tocopherols at 356.49–930.43 mg/kg, phytosterols at 1248.61–2155.24 mg/kg, and total phenolic compounds at 15.85–68.51 mg/kg. The significant differences in the comprehensive scores between the Yunnan walnuts and the walnuts from the other provinces may be due to their different growth environments and genetic diversity. The Henan-produced walnuts had a high crude protein content and are suitable for walnut processing, while the Yunnan-produced walnuts were rich in lipid components, therefore making them perfect for producing high-quality walnut oil. The Shaanxi-produced walnuts had a relatively high total amount of amino acids, which make them suitable for the development of walnut cake meal products. The data obtained in this study contribute to a better understanding of the compositional variations in the major walnut-growing regions of China.

## Figures and Tables

**Table 1:** Physicochemical and oxidative stability indices of walnut oils from China.

	AV(KOH)/ (mg/g)	IV (g I2/100 g Oil)	IP (h)	Polyphenols (mg/kg)	DPPH (µmol/100 g)	FRAP (µmol/100 g)
WS1	0.14 ± 0.00 ^g^	158.74 ± 0.20 ^f^	2.86 ± 0.04 ^ijklm^	26.83 ± 0.30 ^defg^	19.44 ± 0.25 ^h^	47.73 ± 0.23 ^m^
WS2	0.14 ± 0.00 ^g^	153.58 ± 0.00 ^m^	2.62 ± 0.03 ^m^	19.80 ± 0.12 ^k^	20.29 ± 0.24 ^h^	56.27 ± 0.18 ^j^
WS3	0.14 ± 0.00 ^g^	153.53 ± 0.01 ^n^	3.69 ± 0.00 ^ghijk^	30.13 ± 0.23 ^d^	14.37 ± 0.33 ^k^	48.01 ± 0.43 ^m^
WS4	0.42 ± 0.00 ^dc^	159.49 ± 0.02 ^k^	3.08 ± 0.02 ^ghijk^	68.51 ± 0.28 ^a^	11.89 ± 0.56 ^l^	41.83 ± 0.17 ^o^
WS5	0.28 ± 0.00 ^bc^	154.32 ± 0.19 ^d^	3.07 ± 0.03 ^hijk^	25.63 ± 1.44 ^defg^	33.65 ± 0.21 ^c^	87.64 ± 0.27 ^c^
WS6	0.49 ± 0.07 ^ef^	153.87 ± 0.02 ^l^	3.58 ± 0.06 ^cde^	26.96 ± 1.14 ^defgh^	42.31 ± 0.33 ^a^	87.41 ± 0.22 ^c^
WS7	0.21 ± 0.07 ^fg^	159.12 ± 0.19 ^e^	2.90 ± 0.11 ^ijklm^	24.13 ± 1.68 ^ghij^	23.39 ± 0.54 ^g^	34.75 ± 0.22 ^r^
WS8	0.42 ± 0.00 ^dc^	149.21 ± 0.00 ^s^	3.20 ± 0.01 ^efghi^	22.80 ± 0.88 ^hijk^	16.36 ± 0.01 ^j^	43.54 ± 0.13 ^n^
WS9	0.35 ± 0.07 ^de^	155.77 ± 0.01 ^j^	3.54 ± 0.16 ^cdef^	21.00 ± 0.33 ^jk^	41.07 ± 0.41 ^b^	89.32 ± 0.04 ^b^
WS10	0.13 ± 0.00 ^g^	156.97 ± 0.02 ^h^	3.39 ± 0.13 ^efgh^	22.49 ± 1.18 ^hijk^	28.92 ± 0.05 ^f^	60.33 ± 0.03 ^i^
WS11	0.21 ± 0.06 ^fg^	160.06 ± 0.02 ^c^	3.32 ± 0.03 ^efgh^	27.25 ± 0.82 ^defg^	17.99 ± 0.41 ^i^	32.86 ± 0.21 ^s^
WS12	0.77 ± 0.07 ^a^	157.22 ± 0.02 ^g^	3.22 ± 0.03 ^efghi^	28.44 ± 0.84 ^de^	28.71 ± 0.20 ^f^	54.88 ± 0.32 ^k^
WS13	0.14 ± 0.00 ^g^	161.95 ± 0.18 ^a^	2.53 ± 0.04 ^m^	21.57 ± 0.84 ^ijk^	16.31 ± 0.00 ^j^	41.42 ± 0.44 ^o^
WS14	0.49 ± 0.07 ^bc^	152.69 ± 0.04 ^o^	3.40 ± 0.03 ^defgh^	38.94 ± 0.51 ^b^	16.37 ± 0.29 ^j^	39.38 ± 0.37 ^p^
WS15	0.41 ± 0.00 ^dc^	152.33 ± 0.01 ^p^	3.16 ± 0.08 ^fghij^	24.37 ± 0.26 ^ghi^	31.11 ± 0.71 ^de^	84.57 ± 0.51 ^d^
WS16	0.42 ± 0.00 ^cd^	161.21 ± 0.20 ^b^	2.74 ± 0.025 ^klm^	15.85 ± 0.25 ^l^	7.44 ± 0.80 ^m^	24.22 ± 0.30 ^t^
WS17	0.77 ± 0.06 ^a^	160.12 ± 0.21 ^c^	8.41 ± 0.45 ^a^	27.84 ± 0.91 ^def^	30.55 ± 0.12 ^e^	93.97 ± 0.23 ^a^
WS18	0.28 ± 0.00 ^ef^	153.89 ± 0.00 ^l^	2.78 ± 0.14 ^jklm^	21.63 ± 0.88 ^ijk^	31.70 ± 0.66 ^de^	76.93 ± 0.85 ^g^
WS19	0.77 ± 0.07 ^a^	153.23 ± 0.01 ^n^	3.22 ± 0.03 ^efghi^	25.56 ± 1.49 ^defgh^	11.85 ± 0.09 ^l^	37.41 ± 0.06 ^q^
WS20	0.27 ± 0.00 ^ef^	156.47 ± 0.01 ^i^	3.32 ± 0.10 ^efgh^	24.72 ± 1.18 ^fghi^	30.94 ± 0.36 ^de^	79.58 ± 0.72 ^f^
WS21	0.55 ± 0.00 ^b^	162.14 ± 0.01 ^a^	2.93 ± 0.07 ^ijkl^	32.74 ± 1.16 ^c^	31.30 ± 0.02 ^de^	94.15 ± 0.01 ^a^
WS22	0.56 ± 0.00 ^b^	151.41 ± 0.02 ^q^	3.47 ± 0.05 ^defg^	26.99 ± 0.30 ^defg^	17.12 ± 0.39 ^ij^	53.37 ± 0.05 ^l^
WS23	0.21 ± 0.07 ^fg^	154.44 ± 0.01 ^k^	3.51 ± 0.01 ^def^	22.46 ± 1.09 ^hijk^	16.46 ± 0.41 ^j^	44.13 ± 0.08 ^n^
WS24	0.70 ± 0.00 ^a^	145.82 ± 0.02 ^t^	3.90 ± 0.13 ^bc^	20.13 ± 1.74 ^k^	32.01 ± 0.75 ^d^	74.00 ± 0.02 ^h^
WS25	0.55 ± 0.00 ^bc^	145.64 ± 0.01 ^t^	4.01 ± 0.06 ^b^	15.87 ± 1.38 ^l^	31.22 ± 0.07 ^de^	83.61 ± 0.31 ^e^
WS26	0.49 ± 0.06 ^bc^	149.58 ± 0.02 ^r^	3.07 ± 0.00 ^hijk^	25.23 ± 1.08 ^efgh^	7.63 ± 0.23 ^m^	37.85 ± 0.26 ^q^

Values are means ± standard deviation. The superscript letters indicate the statistical difference in columns at a significant level of 5%. AV: acid value; IP: induction period; DPPH: 2,2-diphenyl-1-picrylhydrazyl; FRAP; ferric-reducing antioxidant power.

**Table 2:** Variance contribution ratios of PCA to the quality characteristics of walnut oil from 11 provinces in China.

Component	Initial Eigenvalue			Extraction Sums of Squared Loadings		
	Total	% of Variance	Cumulative %	Total	% of Variance	Cumulative %
1	6.762	29.40	29.40	6.76	29.40	29.40
2	5.87	25.52	54.92	5.87	25.52	54.92
3	2.70	11.76	66.68	2.70	11.76	66.68
4	2.23	9.69	76.37	2.23	9.69	76.37
5	1.69	7.34	83.71	1.69	7.34	83.71
6	1.41	6.14	89.85	1.41	6.14	89.85
7	1.15	4.99	94.85	1.15	4.99	94.85
8	0.62	2.69	97.53			
9	0.36	1.57	99.10			
10	0.21	0.90	100.00			

**Table 3:** A comprehensive evaluation of walnut oils from 11 provinces in China.

Ranking	Growing Location	Score
NO. 1	Henan	2.06
NO. 2	Gansu	1.33
NO. 3	Zhejiang	0.59
NO. 4	Hebei	0.38
NO. 5	Shanxi	0.05
NO. 6	Xinjiang	−0.18
NO. 7	Hubei	−0.39
NO. 8	Sichuan	−0.46
NO. 9	Shandong	−0.50
NO. 10	Shaanxi	−1.13
NO.11	Yunnan	−1.78

## Data Availability

The data used to support the findings of this study can be made available by the corresponding author upon request.

## Supplementary Materials

The following supporting information can be downloaded at: https://www.mdpi.com/article/10.3390/foods12224123/s1. Table S1. Composition and content of the main lipid concomitants in walnut oil.
