# Supplementary material for: Quality Evaluation of Walnuts from Different Regions in China
Source: Foods. 2023 Nov 14;12(22):4123. doi: 10.3390/foods12224123 (PMC10670351; doi:10.3390/foods12224123)
Supplement: Supplementary file 1 [file foods-12-04123-s001.zip › foods-2678662-supplementary.pdf]

Table S1. Composition and content of the main lipid concomitants in walnut oil

	Protein (%)	oil content (%)	C16:0 (%)	C18:0 (%)	C18:1 (%)	C18:2 (%)	C18:3 (%)	SFA (%)	UFA (%)	α- tocopherol （mg/kg）
WS1	19.08±0.05 ^{b}	66.49±0.00 ^{jk}	6.70±0.02 ^{f}	2.82±0.01 ^{h}	17.49±0.26 ^{lm}	61.14±0.20 ^{k}	11.82±0.02 ^{d}	9.52±0.00 ^{f}	90.47±0.10 ^{p}	75.62±1.66^{a}
WS2	16.79±0.02 ^{d}	67.93±0.04 ^{efg}	5.71±0.0 ^{p}	2.67±0.00 ^{l}	22.80±0.00 ^{e}	59.62±0.00 ^{n}	9.20±0.00 ^{l}	8.36±0.00 ^{pq}	91.63±0.00 ^{ef}	69.05±0.33 ^{cdef}
WS3	15.84±0.02 ^{ef}	68.26±0.00 ^{ef}	5.71±0.00 ^{p}	2.66±0.00 ^{k}	22.81±0.00 ^{e}	59.62±0.00 ^{n}	9.18±0.00 ^{l}	8.37±0.00 ^{p}	91.62±0.00 ^{f}	62.38±0.10 ^{ij}
WS4	15.07±0.03 ^{hi}	70.38±0.00 ^{abc}	6.44±0.01 ^{i}	2.32±0.00 ^{r}	24.57±0.21 ^{c}	54.13±0.15 ^{q}	12.52±0.03 ^{c}	8.76±0.00 ^{m}	91.23±0.07 ^{i}	63.23±0.12 ^{hij}
WS5	15.76±0.00 ^{ef}	69.87±0.00 ^{c}	5.67±0.00 ^{q}	2.55±0.00 ^{o}	18.82±0.00 ^{j}	61.55±0.00 ^{j}	11.39±0.00 ^{g}	8.22±0.00 ^{r}	91.77±0.00 ^{d}	67.21±0.27 ^{def}
WS6	15.03±0.16 ^{i}	70.12±0.00 ^{bc}	8.27±0.01 ^{a}	2.36±0.01 ^{q}	17.72±0.04 ^{l}	62.65±0.02^{f}	8.97±0.01 ^{n}	10.64±0.00 ^{a}	89.36±0.01 ^{u}	69.86±1.10 ^{bcde}
WS7	15.31±0.36 ^{ghi}	62.34±0.00 ^{o}	6.52±0.0 ^{h}	2.62±0.0 ^{m}	17.41±0.20 ^{mn}	62.03±0.18 ^{i}	11.40±0.0 ^{g}	9.14±0.00 ^{i}	90.85±0.0 ^{m}	65.88±0.21 ^{fgh}
WS8	16.79±0.04 ^{d}	65.06±0.00 ^{mn}	5.61±0.0 ^{r}	3.09±0.00 ^{d}	25.78±0.00 ^{b}	57.41±0.00 ^{o}	8.08±0.00 r	8.71±0.00 ^{n}	91.28±0.0 ^{h}	72.45±0.04 ^{ab}
WS9	19.50±0.28 ^{a}	66.58±0.00 ^{ijk}	5.92±0.00 ^{n}	2.41±0.00 ^{p}	20.14±0.00 ^{h}	62.62±0.00 ^{f}	8.89±0.00 ^{o}	8.33±0.00 ^{q}	91.66±0.00 ^{e}	70.41±1.27 ^{bcd}
WS10	15.53±0.10 ^{fg}	66.54±0.00 ^{jk}	5.88±0.00 ^{o}	3.01±0.00 ^{f}	19.71±0.00 ^{i}	60.52±0.00 ^{m}	10.86±0.00^{h}	8.89±0.00 ^{l}	91.10±0.00 ^{j}	74.41±1.13 ^{a}
WS11	14.06±0.05 ^{j}	69.10±0.00 ^{d}	6.35±0.00 ^{j}	3.07±0.00 ^{e}	15.93±0.00 ^{pq}	63.11±0.00 ^{e}	11.52±0.00 ^{f}	9.42±0.00 ^{g}	90.57±0.00 ^{o}	72.78±0.77 ^{ab}
WS12	17.38±0.02 ^{c}	64.74±0.00 ^{n}	5.46±0.00 ^{s}	3.44±0.00 ^{b}	17.19±0.00 ^{n}	65.24±0.00 ^{b}	8.65±0.00 ^{p}	8.90±0.00 ^{kl}	91.09±0.00 ^{jk}	71.02±0.32 ^{bc}
WS13	15.10±0.13 ^{ghi}	69.04±0.00 ^{d}	6.20±0.02 ^{k}	2.72±0.01 ^{i}	16.05±0.23 ^{p}	62.30±0.18 ^{g}	12.71±0.02 ^{b}	8.93±0.00 ^{k}	91.06±0.09 ^{k}	66.63±1.78 ^{efg}
WS14	15.89±0.07 ^{ef}	68.64±0.00 ^{de}	6.05±0.00 ^{l}	3.55±0.00 ^{a}	20.73±0.01 ^{g}	61.00±0.01 ^{kl}	8.64±0.00 ^{p}	9.61±0.00 ^{e}	90.38±0.00 ^{q}	67.42±0.53 ^{def}
WS15	16.77±0.03 ^{d}	66.88±0.00 ^{hi}	5.11±0.00 ^{u}	3.13±0.00 ^{c}	23.22±0.00 ^{d}	60.50±0.00 ^{m}	8.02±0.00 ^{r}	8.24±0.00 ^{r}	91.77±0.00 ^{d}	63.76±2.21 ^{ghi}
WS16	15.45±0.01 ^{fghi}	69.07±0.00 ^{d}	6.61±0.01 ^{g}	2.60±0.00 ^{n}	16.35±0.23 ^{o}	61.65±0.17 ^{j}	12.77±0.03 ^{a}	9.21±0.00 ^{h}	90.78±0.08 ^{n}	62.89±0.51 ^{hij}
WS17	7.26±0.00 ^{k}	67.30±0.00 ^{gh}	8.18±0.00 ^{b}	2.31±0.00 ^{r}	18.14±0.00 ^{k}	62.22±9.12 ^{gh}	9.12±0.00 ^{m}	10.50±0.00 ^{b}	89.49±0.00 ^{t}	72.77±0.04 ^{ab}
WS18	16.18±0.01 ^{e}	68.52±0.00 ^{de}	6.02±0.01 ^{m}	2.93±0.00 ^{g}	15.59±0.23 ^{q}	64.88±0.18 ^{c}	10.53±0.02 ^{i}	8.98±0.00 ^{j}	91.01±0.08 ^{l}	58.19±1.18 ^{klm}
WS19	17.66±0.21 ^{c}	66.12±0.00 ^{kl}	6.78±0.00 ^{e}	2.32±0.00 ^{r}	20.12±0.00 ^{h}	63.10±0.00 ^{e}	7.65±0.00 ^{s}	9.11±0.00 ^{i}	90.88±0.00 ^{m}	56.91±0.21 ^{lm}
WS20	15.51±0.03 ^{fgh}	66.39±0.00 ^{jk}	5.27±0.00 ^{t}	3.07±0.00 ^{e}	20.03±0.00 ^{h}	62.05±0.00 ^{hi}	9.56±0.00 ^{k}	8.34±0.00 ^{pq}	91.65±0.00 ^{ef}	55.59±0.26 ^{m}
WS21	15.89±0.25 ^{ef}	68.01±0.00 ^{ef}	5.91±0.00 ^{n}	2.67±0.00 ^{j}	15.50±0.01 ^{r}	64.20±0.01 ^{d}	11.70±0.00 ^{e}	8.59±0.00 ^{o}	91.40±0.00 ^{g}	60.96±0.03 ^{ijk}
WS22	16.97±0.12 ^{d}	67.73±0.00 ^{fg}	7.41±0.00 ^{d}	2.43±0.00 ^{p}	21.14±0.00 ^{f}	60.91±0.00 ^{l}	8.10±0.00 ^{q}	9.83±0.00 ^{d}	90.16±0.00 ^{r}	58.87±0.87 ^{kl}
WS23	14.21±0.13 ^{j}	71.06±0.00 ^{a}	7.84±0.00 ^{c}	2.28±0.00 ^{s}	18.91±0.00 ^{j}	61.19±0.01 ^{k}	9.76±0.00 ^{j}	10.13±0.00 ^{c}	89.86±0.00 ^{s}	60.02±0.25 ^{jkl}
WS24	16.79±0.16 ^{d}	65.50±0.00 ^{lm}	4.69±0.00 ^{v}	2.23±0.00 ^{t}	32.28±0.01 ^{a}	53.44±0.00 ^{r}	7.34±0.00 ^{t}	6.92±0.00 ^{t}	93.07±0.00 ^{b}	62.40±2.78 ^{ij}
WS25	17.69±0.01 ^{c}	60.08±0.00 ^{p}	4.61±0.00 ^{w}	2.17±0.00 ^{u}	32.05±0.00 ^{a}	54.52±0.00 ^{p}	6.63±0.00 ^{u}	6.7830.00 ^{u}	93.21±0.00 ^{a}	60.11±0.26 ^{jk}
WS26	14.15±0.08 ^{j}	70.63±0.00 ^{ab}	5.25±0.00 ^{t}	1.90±0.01 ^{v}	22.73±0.01 ^{e}	67.64±0.00 ^{a}	2.45±0.00 ^{v}	7.16±0.00 ^{s}	92.83±0.00 ^{c}	68.33±1.12 ^{cdef}

Values are means ± standard deviation. The superscript letters indicate the statistical difference in columns at a significant level of 5%. N. D., not

detected. palmitic acid (C16:0), stearic acid (C18:0), oleic acid (C18:1), linoleic acid (C18:2), α-linolenic acid (C18:3), SFA(C16:0+C18:0), UFA (C18:1+ C18:2+C18:

Table S1. *(Continued)*

	γ tocopherol (mg/kg)	δ-tocopherol (mg/kg)	TTC (mg/kg)	Stigmasterol	β-Sitosterol	Fucosterol	Cycloarterol	Erythrodiol	TPC (mg/kg)
WS1	326.52±0.04 ^{n}	137.44±0.14 ^{ef}	539.59± 0.74 ^{n}	51.30±0.33 ^{bcdefg}	1132.30±0.40 ^{h}	153.70±0.14 ^{bc}	282.20±0.07 ^{d}	ND	1619.62±0.29 ^{hijk}
WS2	425.89±3.17 ^{i}	144.33±1.31 ^{de}	639.27±1.18 ^{kl}	57.30±0.37 ^{bcd}	1182.90±1.46 ^{efgh}	145.30±0.33 ^{bcd}	209.60±0.34 ^{f}	ND	1595.33±2.39 ^{ijk}
WS3	376.66±2.63 ^{kl}	124.33±0.97 ^{gh}	563.19±0.65 ^{mn}	64.80±0.10 ^{bc}	1162.80±2.28 ^{fgh}	112.30±0.21 ^{efghi}	202.00±0.41 ^{f}	ND	1542.12±2.65 ^{kl}
WS4	344.10±4.68 ^{n}	123.97±0.17 ^{gh}	531.30±2.14 ^{n}	48.00±0.18 ^{cdefg}	1161.10±1.29 ^{fgh}	139.10±0.39 ^{bcde}	341.00±0.40 ^{b}	ND	1689.33±1.79 ^{efgh}
WS5	491.63±1.94 ^{gh}	110.78±0.55 ^{jkl}	669.63±0.73 ^{jk}	57.90±0.28 ^{bcd}	1248.50±2.18 ^{cde}	129.60±0.01 ^{cdefg}	178.60±0.01 ^{g}	540.50±1.99 ^{b}	2155.24±4.48 ^{a}
WS6	581.63±3.86 ^{c}	110.65±0.85 ^{jkl}	762.14±1.36 ^{ef}	41.15±0.27 ^{defg}	1073.40±1.06 ^{i}	92.90±0.22 ^{ijkl}	239.00±0.10 ^{e}	607.20±4.17 ^{a}	2053.81±2.97 ^{b}
WS7	394.95±2.5 ^{m}	156.08±0.46 ^{bc}	616.91±1.05 ^{l}	64.40±0.02 ^{bc}	1240.70±5.58 ^{cde}	154.20±0.66 ^{bc}	330.50±1.43 ^{bc}	48.60±0.16 ^{d}	1838.60±7.86 ^{cd}
WS8	506.76±3.46 ^{fgh}	172.80±4.44 ^{a}	752.02±1.88 ^{fg}	108.80±3.77 ^{a}	162.60±8.31 ^{fgh}1	158.00±5.42 ^{b}	36.70±1.26 ^{k}	ND	1466.22 ±2.14 ^{l}
WS9	486.22±1.83 ^{h}	159.00±1.46 ^{bc}	715.62±0.23 ^{ghi}	57.40±0.26 ^{bcd}	1211.30±1.46 ^{defg}	123.80±0.25 ^{defgh}	199.20±0.21 ^{f}	ND	1591.74 ±1.67 ^{ik}
WS10	516.96±2.59 ^{f}	120.90±1.90 ^{ghi}	714.28±0.60 ^{ghi}	54.00±1.03 ^{bcdef}	1179.50±1.65 ^{efgh}	135.80±0.33 ^{bcdef}	239.40±0.44 ^{e}	ND	1608.91±2.53 ^{hijk}
WS11	552.07±4.99 ^{d}	109.42±0.13 ^{jkl}	752.02±1.88 ^{fgh}	41.10±0.15 ^{defg}	1188.60±3.66 ^{efgh}	115.10±0.46 ^{efghi}	337.40±0.86 ^{bc}	ND	1682.30±4.72 ^{fghi}
WS12	527.21±2.44 ^{ef}	103.68±0.11 ^{kl}	701.91±1.05 ^{hij}	67.00±0.24 ^{bc}	1429.00±1.97 ^{ab}	107.60±0.12 ^{ghi}	154.60±0.16 ^{i}	ND	1758.32 ±2.46^{def}
WS13	362.11±1.32 ^{lm}	151.61±1.71 ^{cd}	580.35± 0.20 ^{m}	55.10±0.69 ^{bcde}	1215.07±0.90 ^{defg}	146.10±0.23 ^{bcd}	324.10±0.61 ^{c}	ND	1740.45±1.07 ^{efg}
WS14	401.79±0.09 ^{j}	109.32±1.16 ^{jkl}	578.53±0.26 ^{m}	70.90±0.59 ^{b}	1374.10±3.02 ^{b}	96.20±0.63 ^{hijk}	206.00±0.82 ^{f}	276.50±2.60^{c}	2023.84±6.24 ^{b}
WS15	684.48±9.36 ^{b}	115.94±4.18 ^{hij}	864.18±3.02 ^{d}	62.10±1.28 ^{bcd}	1297.50±3.80 ^{c}	102.60±0.74 ^{ghij}	197.10±0.79 ^{f}	ND	1659.44±5.80 ^{ghij}
WS16	557.52±29.28 ^{d}	174.41±4.01 ^{a}	794.82±12.81 ^{de}	47.20±0.19 ^{cdefg}	1187.30±1.91 ^{efgh}	162.40±0.29 ^{b}	359.90±0.48 ^{a}	ND	1756.92±2.69 ^{def}
WS17	539.19±1.49 ^{de}	111.73±1.82 ^{ijkl}	723.69±0.77 ^{fghi}	32.30±0.40 ^{fg}	1229.80±6.26 ^{def}	443.20±2.25 ^{a}	149.10±0.73 ^{i}	58.10±0.21 ^{d}	1979.11±0.12 ^{c}
WS18	298.30±3.29 ^{o}	ND	356.49±1.06 ^{o}	60.10±1.21 ^{bcd}	1260.20±1.68 ^{cd}	114.10±1.22 ^{efghi}	338.90±0.14 ^{bc}	ND	1773.32±3.63 ^{de}
WS19	551.75±3.68 ^{d}	115.24±0.29 ^{hijk}	723.90±1.62 ^{fghi}	68.50±1.62 ^{bc}	1157.20±1.05 ^{gh}	76.50±0.34 ^{jkl}	171.30±1.16 ^{gh}	ND	1473.69±1.50 ^{l}
WS20	408.73±0.56 ^{ij}	103.73±1.15 ^{kl}	568.05±0.37 ^{mn}	47.60±0.11 ^{cdefg}	1471.60±1.27 ^{a}	153.00±0.17 ^{bc}	160.10±0.41 ^{hi}	ND	1832.30±1.14 ^{cd}
WS21	332.18±4.05 ^{n}	141.00±1.60 ^{de}	534.14±1.65 ^{n}	41.30±0.36 ^{defg}	1197.00±2.10 ^{defgh}	109.00±0.07 ^{fghi}	269.50±0.35 ^{d}	ND	1616.90±2.44 ^{hijk}
WS22	707.45±2.26 ^{a}	127.84±1.61 ^{fg}	894.16±0.57 ^{b}	31.60 ±0.13 ^{g}	981.25±1.58 ^{j}	79.20±0.19 ^{jkl}	156.50±0.51 ^{hi}	ND	1248.61±2.29 ^{n}
WS23	705.53±5.39 ^{a}	164.88±0.22 ^{ab}	930.43 ±2.43 ^{a}	34.20±0.65 ^{efg}	1238.30±1.28 ^{cde}	73.70±1.09 ^{kl}	239.40±0.33 ^{e}	ND	1585.82±0.99 ^{jk}
WS24	583.08±2.97 ^{c}	102.78±0.62 ^{l}	748.26±1.07 ^{fg}	58.70±0.26 ^{bcd}	1465.60±0.51 ^{a}	76.40±0.65 ^{jkl}	80.60±1.63 ^{j}	ND	1681.44±1.86$^{f\ ghi}$
WS25	508.15±3.24 ^{fg}	118.59±16.71 ^{ghij}	686.84±7.15 ^{ij}	34.80±0.31 ^{efg}	1139.90±0.21 ^{h}	97.4±0.14 ^{hijk}	83.30±1.38 ^{j}	ND	1355.60±1.52 ^{m}
WS26	667.71±11.96 ^{b}	119.94±1.75 ^{ghij}	855.98±4.97 ^{c}	40.10±0.53 ^{defg}	930.80±0.00 ^{j}	67.40±1.12 ^{l}	228.00±0.31^{e}	ND	1266.40±1.96 ^{n}

Values are means ± standard deviation. The superscript letters indicate the statistical difference in columns at a significant level of 5%. N. D., not detected. TTC: Total tocopherol content, TPC: Total phytosterol content:
